# Multimodal Imaging Signatures of Parkinson's Disease

**DOI:** 10.3389/fnins.2016.00131

**Published:** 2016-04-18

**Authors:** F. DuBois Bowman, Daniel F. Drake, Daniel E. Huddleston

**Affiliations:** ^1^Department of Biostatistics, The Mailman School of Public Health, Columbia UniversityNew York, NY, USA; ^2^Department of Neurology, Emory UniversityAtlanta, GA, USA

**Keywords:** multimodal imaging, MRI, prediction, classification, penalized regression, Parkinson's disease, biomarker

## Abstract

Parkinson's disease (PD) is a complex neurodegenerative disorder that manifests through hallmark motor symptoms, often accompanied by a range of non-motor symptoms. There is a putative delay between the onset of the neurodegenerative process, marked by the death of dopamine-producing cells, and the onset of motor symptoms, creating an urgent need to develop biomarkers that may yield early PD detection. Neuroimaging offers a non-invasive approach to examining the potential utility of a vast number of functional and structural brain characteristics as biomarkers. We present a statistical framework for analyzing neuroimaging data from multiple modalities to determine features that reliably distinguish PD patients from healthy control (HC) subjects. Our approach builds on elastic net, performing regularization and variable selection, while introducing additional criteria centering on parsimony and reproducibility. We apply our method to data from 42 subjects (28 PD patients and 14 HC). Our approach demonstrates extremely high accuracy, assessed via cross-validation, and isolates brain regions that are implicated in the neurodegenerative PD process.

## Introduction

**Figure 1:**
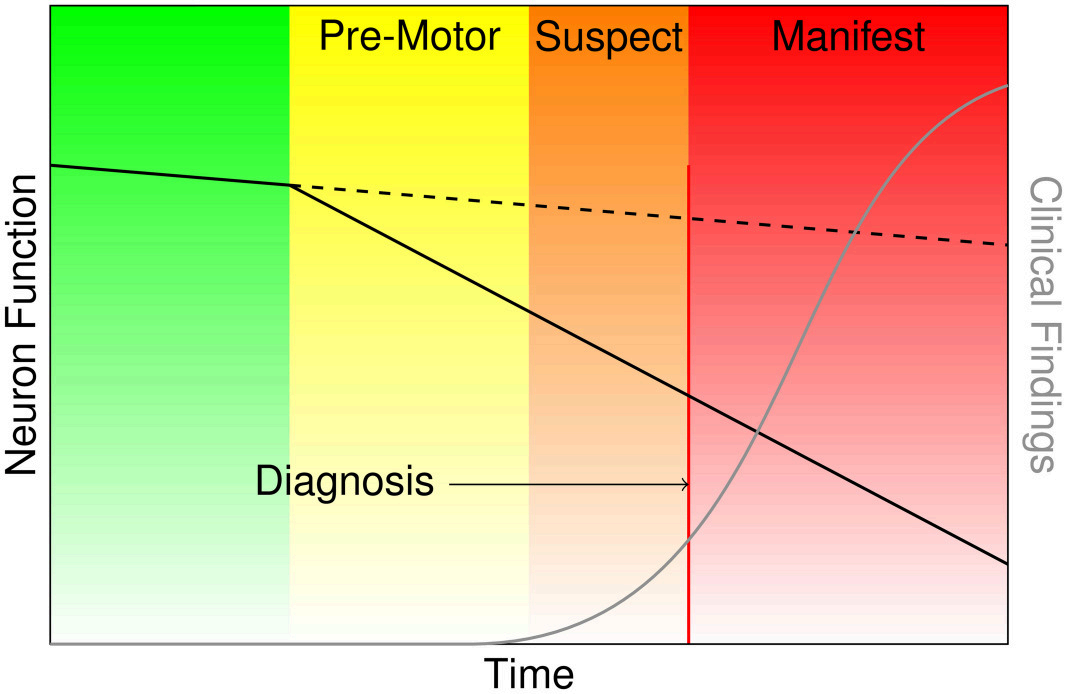
**Characterization of the onset and progression of Parkinson's disease neurodegeneration (green and yellow), which persists through the commencement of motor symptoms (orange) and ultimately clinical diagnosis and beyond (red)**.

Parkinson's disease (PD) is a devastating, progressive movement disorder affecting 7–10 million individuals worldwide (Parkinson's Disease Foundation, 2015). PD usually affects people over 50 years of age, but a subset of patients experience early onset. The hallmark pathology of PD is the loss of dopaminergic neurons in the substantia nigra pars compacta (SNpc), but the disease manifests with a diversity of symptoms referable to multi-system neuropathology. The clinical features of PD include the classic motor symptoms of tremor, rigidity, bradykinesia, and gait impairment, as well as a host of non-motor symptoms (Kalia and Lang, 2015). At the time of PD diagnosis it has been estimated based on histopathology that over 50% of dopamine neurons in the SNpc have died (Fearnley and Lees, 1991). Braak et al. (2003) posit a process of phased pathology of PD, which suggests that early neurodegeneration occurs in lower brainstem structures and progresses in ascending fashion, in particular affecting the locus coeruleus in Stage II and SNpc in Stage III. Further progression extends to higher-level sensory association areas and prefrontal cortical regions, eventually impacting first order sensory association areas, premotor regions, and primary sensory and motor fields (Del Tredici and Braak, 2013). The putative delay in the onset of motor symptoms leading to PD diagnosis is portrayed in Figure 1, and the corresponding neurodegeneration occurring throughout this pre-motor period represents a missed opportunity for early therapeutic intervention that may significantly slow or halt the progression of PD related decline.

There is so far no reliable method to accurately diagnose PD in its pre-motor stages, and addressing this unmet need is a key challenge in the field of PD biomarker development. Many pre-motor symptoms of PD are non-specific, including depression, anxiety, constipation, and excessive daytime sleepiness (Tolosa and Pont-Sunyer, 2011). REM sleep behavior disorder (RBD) in the absence of dementia, hallucinations, autonomic dysfunction or parkinsonian motor symptoms, referred to as idiopathic RBD (iRBD), portends a high likelihood of eventual conversion to a synucleinopathy: PD, multiple system atrophy or Lewy body dementia (Iranzo et al., 2013). However, simply identifying iRBD does not allow prediction of the specific clinical phenotype a patient will develop, and the duration to phenoconversion is variable from the time of iRBD diagnosis (Iranzo et al., 2013; Postuma et al., 2015), which makes pre-motor PD study design in this group more challenging. Furthermore, clinical presentation with iRBD prior to evidence of a broader neurodegenerative syndrome is also relatively uncommon and most PD patients have not sought treatment for iRBD prior to phenoconversion with motor symptoms. Other strategies for pre-motor diagnosis of PD have included combining clinical features, such as olfactory loss and family history, with dopamine transporter radionuclide imaging (The Parkinson At-Risk Study or PARS), or an algorithmic approach to develop a cohort enriched with an at-risk genotype, such as LRRK2 G2019S mutation (Tolosa and Pont-Sunyer, 2011; Foroud et al., 2015). While it appears likely that a multi-tiered screening process to identify pre-motor or asymptomatic at risk subjects has promise, inclusion of neuroimaging in a cost-effective manner for in vivo confirmation of PD associated brain pathology may speed up and improve the efficiency of these studies. MRI is a fraction of the cost of radionuclide imaging (Fiandaca et al., 2014), and allows efficient collection of multiple types of disease-relevant brain measurements, including assessment of structural and functional connectivity, which are expected to be impacted by the degeneration of the widely projecting catecholamine neurons affected in PD. Here we leverage advanced statistical methods to identify robust candidate biomarkers and profiles from a large number of MRI features to differentiate patients with early to moderate PD from controls. Because the neurodegeneration process is already advanced at the time of PD diagnosis, a highly robust biomarker in early to moderate (motor) PD patients is likely to be detectable in the pre-motor state as well. Therefore, the outputs of this study may serve as candidate neuroimaging biomarkers in future studies of pre-motor or asymptomatic PD.

Our research is driven by a broad initiative called the Parkinson's Disease Biomarker Program (PDBP) at the National Institutes of Health's (NIH's) National Institute of Neurological Disorders and Stroke to identify early stage biomarkers for PD. In the context of our study, we regard biomarkers in a general sense, defined by an NIH Biomarkers Definitions Working Group as “a characteristic that is objectively measured and evaluated as an indicator of normal biological processes, pathogenic processes, or pharmacologic responses to a therapeutic intervention,” although more specific molecular definitions have been proposed (Strimbu and Tavel, 2010). A first step in identifying early stage PD neuroimaging biomarkers is to determine neural characteristics that reliably distinguish patients with mild to moderate PD from healthy control subjects.

Neuroimaging has shown early promise for identifying alterations associated with PD. There is emerging evidence of cortical thinning in PD patients determined from T1 MRI (Lee et al., 2013; Zarei et al., 2013; Zhang et al., 2015). Vaillancourt et al. (2009) established neuroimaging correlates of PD through decreases in fractional anisotropy generated from diffusion tensor imaging (DTI) data within caudal regions of the substantia nigra. Du et al. (2011) found that augmenting fractional anisotropy measures of the substantia nigra with its transverse relaxation rate, R2^*^, improved the discrimination of PD patients from controls over that of using fractional anisotropy alone. Kahan et al. (2014) target effective connectivity in PD using resting state fMRI (rs-fMRI) in patients with deep brain stimulation, suggesting that subthalamic nucleus modulates major components of the motor cortico-striato-thalamo-cortical loop.

Single modality neuroimaging renders only a partial view toward understanding the neural basis for PD. When targeting classification or prediction, simultaneously examining data from multiple imaging modalities stands to increase accuracy, to provide a more complete picture of the multiple neuropathophysiologic manifestations of PD, and to determine the relative predictive strengths of the PD-related functional and structural changes.

We conduct a novel multimodal imaging investigation that seeks to identify functional and structural changes in mild to moderate PD, which collectively yield high prediction accuracy in dissociating patients from healthy control subjects. Of note, our goals extend beyond simply achieving high prediction accuracy. We aim to contribute to PD biomarker discovery efforts by determining the potential involvement of specific brain regions in the disease process, whether novel or previously studied, which will help to direct future research. Therefore, we balance our objective of high accuracy with criteria of parsimony and reproducibility.

We utilize elastic net, an advanced statistical learning technique, building in novel refinements to enhance performance and to achieve desired levels of parsimony and reproducibility. Elastic net blends both L_1_ and L_2_ penalties, applied here in context of logistic regression, to perform both regularization and variable selection (Zou and Hastie, 2005). We apply the analysis techniques to a set of measures based on magnetic resonance imaging (MRI), including structural T1 images, rs-fMRI, and DTI from PD patients and healthy control subjects. We perform cross-validation to assess accuracy. Overall, the approach achieves extremely high accuracy and reveals key neuroimaging contributors that help to reliably distinguish PD patients from healthy controls.

## Experimental data and methods

### Experimental data

All subject records and data, collected under the auspices of a previous study, were supplied de-identified, stripped of any protected health information (PHI) and personally identifiable information (PII). Accordingly, this research qualifies as Research of Existing Data, Records, Specimens [Basic Exempt Criteria 45 CFR 46.101(b)(4)], and has been deemed “Not Human Subjects Research” (HS Code 10 in IPMAC II as referenced in the manual chapter 7410) by NIH and Columbia University Medical Center Institutional Review Board (Protocol: IRB-AAAO0062).

We consider data from 42 subjects, including 28 PD patients and 14 healthy control (HC) subjects. The data include a collection of magnetic resonance (MR) derived scans characterizing different structural and functional properties of the brain as well as demographic measures. Specifically, we use T1- weighted anatomical MRI scans, rs-fMRI, and DTI. The mean age of the subjects is 65.0 years (9.0 years standard deviation), and the subjects include 21 males and 21 females. The mean age is 61.9 years (8.7 years standard deviation) for PD patients and 71.4 years (5.8 years standard deviation) for controls (a significant difference, with *p* < 0.001). The PD group has 13 females (46.4% of PD patients) and the control group has 8 females (57.1% of controls), reflecting a small sex difference between groups, although not statistically significant (*p* = 0.74). The mean Unified Parkinson's Disease Rating Scale (UPDRS) Part III (motor) score for these patients was 19.4 (standard deviation 10.2). The mean duration of disease was 7.7 years (standard deviation 3.3 years), although the duration was not calculable for 5 patients due to missing data.

All scans were captured with a Siemens Trio Tim 3T MRI scanner; the first 36 subjects were scanned with a 12 channel head coil and the remaining 6 subjects (5 PD and 1 control) were scanned with a 32 channel head coil (we control for this difference in the statistical analyses). The structural T1 scans were acquired using MPRAGE (TR = 2600 ms, TE = 3 ms, 192 sagittal slices at 1 mm; 256 × 232 1 mm isotropic pixels). Echo planar imaging (EPI) was used to acquire 140 frames of rs-fMRI scans (TR = 3000 ms, TE = 30 ms, 48 axial slices at 3 mm, 128 × 128 2 mm isotropic pixels) for each subject. DTI data were captured using a biphase approach with consecutive left-to-right and right-to-left phase scans. The first thirty six subjects underwent DTI scans (TR = 8700ms, TE = 94ms, 64 axial slices at 2 mm, 128 × 128 2 mm isotropic pixels) comprised of 64 directions (B = 1000s/mm^2^), with three leading and three trailing B0 scans. The remaining 6 subjects followed a DTI protocol (TR = 3292 ms, TE = 97.6 ms, 66 axial slices at 2 mm, 92 × 106 2 mm pixels) comprised of 128 directions (B = 1000s/mm^2^), with six leading and five trailing B0 scans.

We implemented standard neuroimaging preprocessing steps including voxel-based morphometry (VBM) on the anatomical T1 scan, using the VBM toolbox (Gaser, 2010) under SPM8, produced voxel-wise estimates of gray matter density in MNI space, along with subject-specific native-to-MNI DARTEL transformations (and their inverses) and gray matter, white matter, and cerebral spinal fluid segmentations. The inverse transformations were used to map MNI-defined parcelations back to each subject's native space. Resting state preprocessing, performed with AFNI (Cox, 1996), consisted of a despiking stage, slice time correction, motion correction, spatial normalization to MNI and smoothing by 6mm FWHM. The resulting rs-fMRI time courses were orthogonalized relative to Legendre polynomials orders 0 through 3; motion parameters and their derivatives; and global white matter and ventricular cerebral spinal fluid (CSF) signals. Finally, the time courses were filtered to the band 0.01–0.1 Hz.

A *t*-test applied to the resting state scans shows no difference in mean temporal SNR between PD (54.4 ± 2.9) and control (53.9 ± 4.9) subjects (*p* = 0.92), with standard error of the mean used to express variability. Similarly, a Wilcoxon rank sum test shows no significant difference in the maximum absolute displacement over the duration of the scan between PD (1.67 mm ± 0.21 mm) and control (1.31 mm ± 0.17 mm) subjects (*p* = 0.53). Finally, another motion-related quantity, the average motion per TR, also does not differ significantly between PD (0.10 mm ± 0.01 mm) and control (0.08 mm ± 0.01 mm) subjects (*p* = 0.38).

For DTI scans, each subject's two opposing phase DTI scans were combined to estimate the susceptibility-induced off-resonance field using a method similar to that described in Andersson et al. (2003) as implemented in FSL (Smith et al., 2004) and the two images were combined into a single corrected one. The resulting composite scan was corrected for eddy currents. After preprocessing, we have 121 × 145 × 121 voxels (1.5 mm isotropic) for DTI and VBM and 91 × 109 × 91 (2 mm isotropic) for rs-fMRI.

### Methods

#### Modality-specific data representations

**Figure 2:**
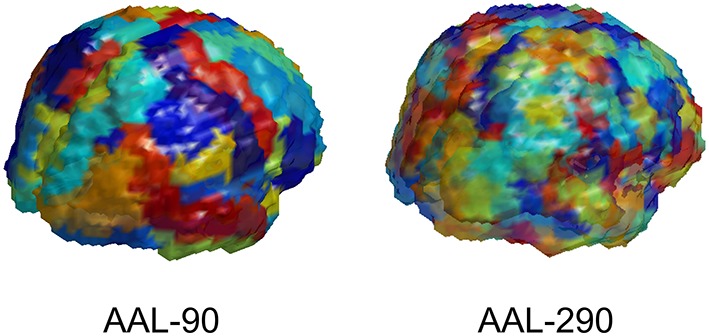
**Depiction of AAL-90 parcellation and a hierarchical subparcellation with 290 brain regions**. The subregions are constructed from resting state fMRI data of healthy controls (outside of the current sample) based on functional characteristics with anatomical constraints to keep subregions contiguous and bounded within a single region.

The first step is to determine the spatial scale for data representations. The imaging data from MRI, rs-fMRI, and DTI are acquired at a voxel level. We utilize a popular neuranatomic parcellation of the brain, the Automated Anatomical Labeling (AAL) (Tzourio-Mazoyer et al., 2002) system, to define 90 brain regions. For MRI and rs-fMRI, we further refine the standard AAL parcellation by defining subregions to yield more homogeneous collections of voxels within subregions. This refinement of the AAL-90 parcellation uses a hierarchical clustering algorithm to subdivide each region based on a metric that combines distance, structural and functional connectivity, and tissue type to identify homogeneous subregions of the encompassing region. The resulting extended parcellation produces 290 subregions (AAL-290), with a given subregion falling entirely within a single AAL region. The regional parcellations appear in Figure 2.

**Figure 3:**
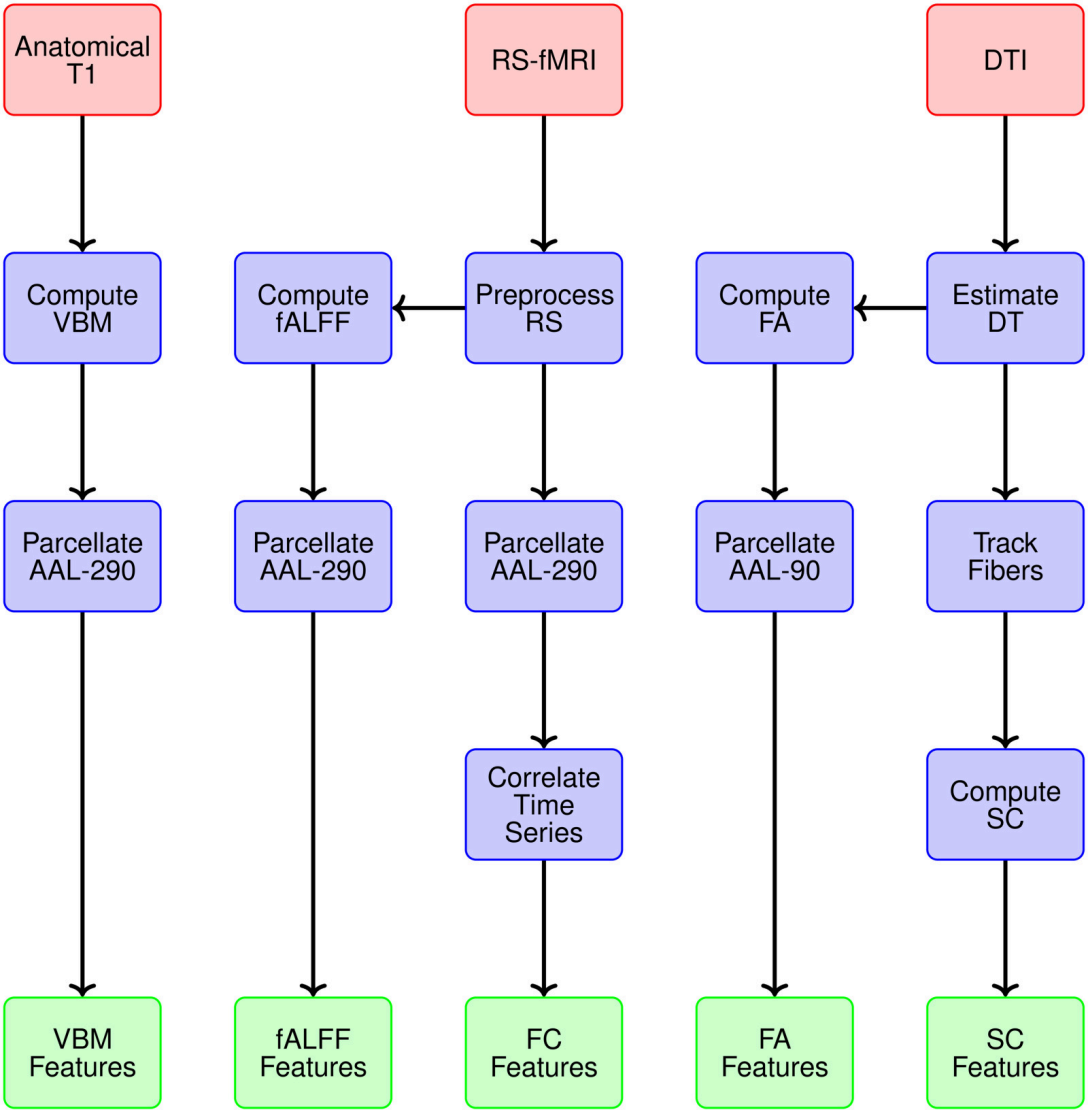
**Overview of the multiple modalities generating data, estimates obtained from each reflecting particular structural or functional properties, spatial scale for summary data representations, and ultimately the features constituting the global set of potential neuroimaging markers of PD**.

We generate data representations (or features) for each imaging modality and specify the spatial scale. Figure 3 provides a conceptual overview describing the multiple modalities generating data, estimates obtained from each reflecting particular structural or functional properties of the brain, the spatial scale for each summary, and ultimately the features constituting the global set of potential neuroimaging markers of PD. We use 290 regions from the extended AAL map (AAL-290) to compute regional averages of local volumetric MRI measures, specifically from voxel-based morphometry (VBM) (see Table 1). We use rs-fMRI data to generate both localized and connectivity features. To quantify the power concentrated at low frequencies for fMRI data, we use fractional amplitude of low frequency fluctuation (fALFF), which calculates the ratio of the power spectrum at low-frequencies (0.01–0.10 Hz) to that of the entire frequency range (Zou et al., 2008). We compute fALFF at a voxel level, for all voxels, and average within each of the 290 subregions. We quantify functional connectivity (FC) by calculating pairwise correlations between the average time courses within each pair of the 290 subregions. We compute fractional anisotropy (FA) for each voxel and obtain regional summaries by averaging over each of the AAL-90 regions. Thus our summary measure will increase both as a function of the restricted diffusion in the regional white matter and the proportion of white matter within a region. We calculate structural connectivity (SC) derived from DTI, using anisotropy to constrain tracking. We use FSL to perform estimation of the diffusion tensor (BEDPOSTX) and tractography (PROBTRACKX) (Behrens et al., 2007).

**Table 1:** **Description of modalities, corresponding features, spatial scale, and screening-based dimension reduction**.

**Modality**	**Feature**	**Spatial Scale**	**Number of Features**	**Post-screening**
MRI	VBM	AAL-290	290 regions	24
rs-fMRI	fALFF	AAL-290	290 subregions	6
rs-fMRI	FC	AAL-290	41,905 subregion pairs	225
DTI	FA	AAL-90	90 regions	6
DTI	SC	AAL-90	4005 region pairs	10
Total			46,580	271

We perform marginal screening to reduce the 46,580 features prior to analysis by eliminating features that are unlikely to carry strong predictive power. Screening typically improves the performance and facilitates implementation of subsequent modeling by eliminating sources of noise and reducing data dimensionality. Toward our goal of attaining reproducible PD biomarkers, we perform a bootstrap screening procedure for each feature independently using logistic regression, with modality- specific screening thresholds. The bootstrap procedure isolates a set of viable markers, after accounting for sampling variability, which increases the likelihood that the identified features will emerge in other samples. Specifically, our screening rule selects features satisfying the following:

$$p^{*}=\frac{1}{B}\sum_{b=1}^{B}I[p_{b}<p_{0}]\geq r.$$

In our analysis, we perform independent screening within each of *B* = 100 bootstrap samples indexed by *b* = 1, …, *B* to obtain a corresponding *p*-value *p*_*b*_, apply designated modality-specific thresholds *p*_0_, and determine features that are selected in at least *r* = 0.75 proportion of the bootstrap samples. After our screening process, we retained 24 regional VBM features (*p*_0_ = 0.2), 6 fALFF (*p*_0_ = 0.2), 225 FC estimates across the brain (*p*_0_ = 0.05), 6 regional FA measures (*p*_0_ = 0.2), and 10 SC estimates (*p*_0_ = 0.2), giving 271 features in total (Table 1).

#### Statistical learning and prediction methods

We propose an analytic approach that uses imaging data from multiple modalities and demographic information to classify subjects as either PD patients or HCs. We present an approach that builds on elastic net with refinements to encourage parsimony and reproducibility. Let *D*_*i*_ = 1, if the *i* th subject has PD and *D*_*i*_ = 0, if subject *i* is a healthy control, *i* = 1, …, *n*. The predictors and an intercept term are arrayed in a vector ${\text{X}}_{i}={\left(1,X_{i1},\ldots ,X_{ip}\right)}^{\prime }$, with *p* denoting the number of predictors following screening. We standardize each of the predictors so that $\sum_{i=1}^{n}x_{ij}=0$ and $(1/n)\sum_{i=1}^{n}x_{ij}^{2}=1$. We let π_*i*_ = Pr(*D*_*i*_ = 1|***X***_*i*_) represent the probability that subject *i* has PD, given a set of predictors, and use logistic regression to model $\log\left[\pi_{i}∕\left(1-\pi_{i}\right)\right]={{\text{X}}_{i}}^{\prime }\beta$. The elastic net procedure applied to logistic regression maximizes the likelihood function

$$\begin{array}{l} \underset{\beta }{\max}\;\{\frac{1}{n}\sum_{i=1}^{n}[D_{i}\log(\pi_{i})+(1-D_{i})\log(1-\pi_{i})]-\\ \;\lambda \sum_{j=0}^{p}\left[\frac{1}{2}\left(1-\alpha \right)\beta_{j}^{2}+\alpha |\beta_{j}|\right]\}. \end{array}$$

From the large set of variables, the method performs shrinkage and variable selection by blending ridge-regression (α = 0) using an L_2_ penalty and the lasso (α = 1) using an L_1_ penalty (Zou and Hastie, 2005). The parameters α and λ are determined by optimizing an objective function via cross-validation, e.g., minimizing the cross-validation error.

The penalized framework, implemented here in context of a logistic model, points to the predictive ability of a specific subset of imaging and demographic variables that constitute a signature for PD in our sample. Ridge regression shrinks the coefficients and tends to draw the coefficients of correlated predictors towards each other. The lasso tends to pull many coefficients near zero, with a small subset of coefficients with larger magnitudes, therefore serving as a useful tool for variable selection. We perform covariate adjustment for demographic variables (age and sex) and scan differences (head coil) in our models. The elastic net penalty is particularly useful when *p* ≫ *n*, and when the set of predictors includes some highly correlated variables, which poses a challenge for L_1_ penalization alone.

We modify the usual optimization procedure for the tuning parameters, when necessary, to promote parsimony, accuracy, and reproducibility (see Results section for details). Our procedure defines a restricted or bounded tuning parameter space, *B*, in which to optimize (α, λ). Specifically, we consider

$$B=\left\{\left(\alpha ,\lambda \right)\;|\;p\leq p_{1},\;AUC\geq q_{1}\right\},$$

where AUC represents the area under the receiver operating characteristic (ROC) curve. Inducing parsimony may sacrifice accuracy, so the subspace *B* incorporates a lower bound on AUC as a measure of accuracy.

We evaluate accuracy using an iterated *k*-fold cross validation scheme for model training and testing to promote reproducibility. A typical implementation of *k*-fold cross validation splits the data into *k* groups, trains the model by fitting the data from *k* − 1 groups (training set), and uses the estimates obtained to predict the disease status of each subject in the remaining group (validation set). The process then rotates the training sets and validation sets until testing has been performed on each group, hence each subject. Variability is inherent in *k*-fold cross validation, which is not typically accounted for in practice. For example, by constructing the folds differently, one may obtain a different estimate of accuracy and detect the involvement of different predictors. To encourage the identification of neuroimaging markers of PD that are reproducible and have high predictive strength and to account for variability in the cross-validation process, we implement an iterated framework. Specifically, we implement two-fold cross-validation and repeat the process 100 times, randomly assigning subjects to folds in each iteration. This process results in 200 training samples.

The cross-validation approach presents an important advantage in the context of our quest to identify likely PD biomarkers, allowing us to gauge the overall importance of each feature by virtue of its average predictive effect. Since the features were standardized, coefficient strengths are comparable: a larger average coefficient strength indicates a greater predictive effect. At each (α, λ) in *B*, we aim to select the top 10% of features based on these coefficient strengths. Let *M* be the random variable representing the magnitude of a predictive effect at (α, λ). Conceptually, imaging features β_*j*_ satisfying Pr(*M* ≥ |β_*j*_|) ≤ τ_*S*_, and which contribute to high predictive accuracy, are regarded as strong candidates for potential biomarkers. In practice, we use the cross-validation process to estimate the empirical distribution function of *M* and determine predictors that have the most sizable effects (on average) across 200 training samples. So for our data, we specifically seek to determine the features satisfying $\bar{\left|\beta_{j}\right|}\geq \xi_{0.10}$, where $\bar{\left|\beta_{j}\right|}$ is the average magnitude of the *j* th effect and ξ_0.10_ is defined by Pr(*M* ≥ ξ_0.10_) = 0.10.

Moreover, we track the consistency with which these predictors with sizable effects are selected for specific values (α, λ) and ultimately choose features that are consistently strong across various combinations (α, λ). Let *S*(α, λ) represent the set of features satisfying the above condition for coefficient strength, i.e., $S(\alpha ,\lambda )=\{\beta_{j}|\bar{\left|\beta_{j}\right|}\geq \xi_{0.10}$ and Pr(*M* ≥ ξ_0.10_) = 0.10;(α, λ)}. Our procedure selects features

$$C=\left\{\beta_{j}|\left[\frac{1}{\#\left[B\right]}\sum_{(\alpha ,\lambda )\in B}I\left[\beta_{j}\in S(\alpha ,\lambda )\right]\right]\geq \tau_{C}\right\},$$

where the notation #[*B*] denotes the cardinality or number of elements in set *B*, and *I* is the indicator function. We set τ_*C*_ = 0.90, effectively taking the features that were selected to be in set *S*(α, λ) in 90% or more of the points in *B*. The set of features in *C* are deemed to have high predictive strength, to be extremely parsimonious, to have high likelihood of emerging in other samples, and to be robust over a range of values in the tuning parameter space. These properties aid the delivery of potential PD biomarkers that can be investigated further in future research. In the application of our methods to the multimodal imaging data of PD patients and healthy controls discussed below, we explore further reductions of the set *C*.

## Results

We applied the methods above to our multimodal imaging data. We consider a 51 × 151 grid of elastic net tuning parameters, with α ∈ [0, 1] and λ ∈ [10^−5^, 10^1^], with 25 points per decade. For every (α, λ) pair, we fit elastic net to half the subjects, then apply the resulting model to the other half of the subjects to predict their disease status. We control for head coil, sex, and age in the model fit. Then we swap sets of subjects and perform the operation again; i.e., two-fold cross validation. We compare the result of the predictions with true disease status to compute the ROC curve and associated AUC value. Finally, we perform this operation 100 times at every (α, λ) in the grid and record the average AUC and various statistics on the model coefficients for each of the 271 features.

**Figure 4:**
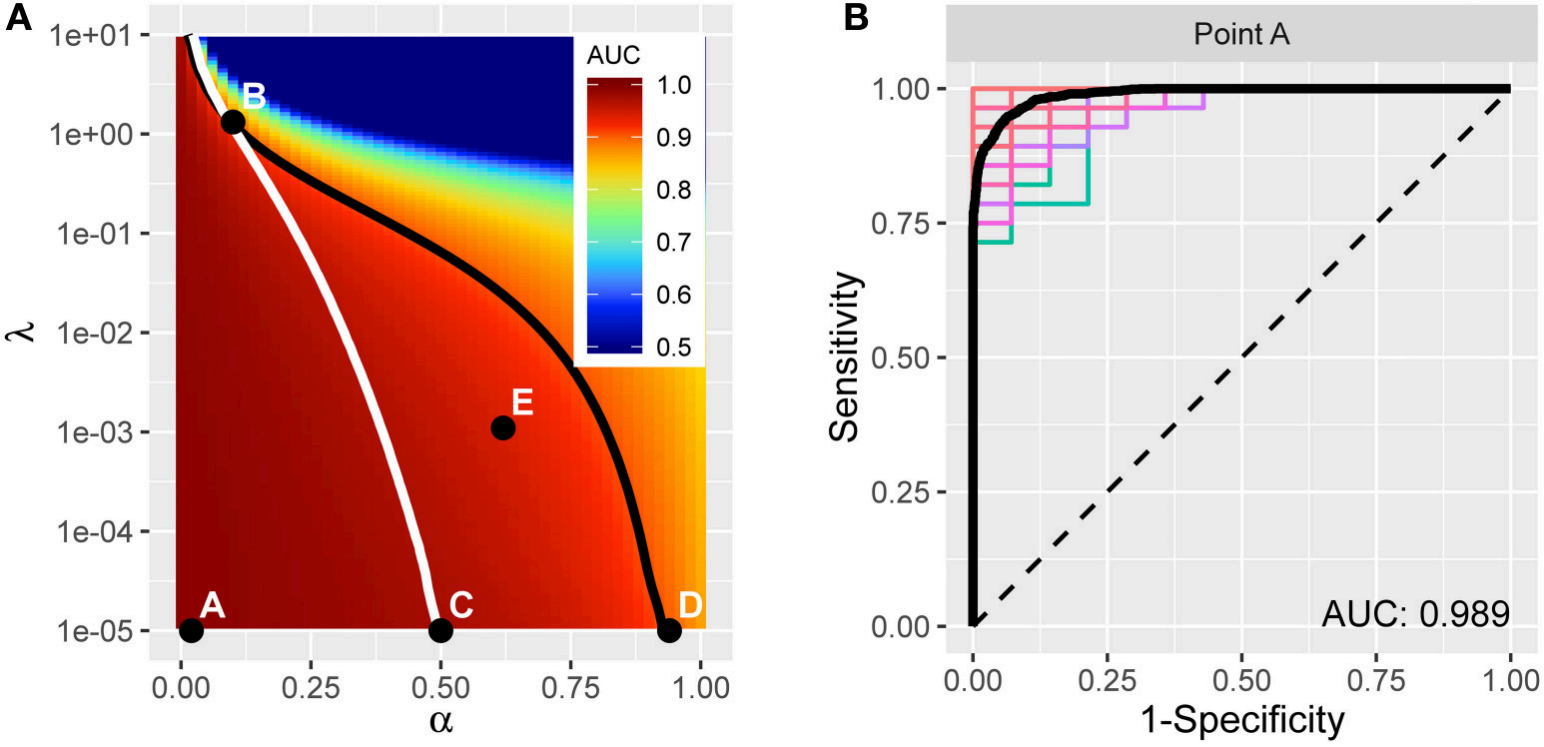
**(A)** AUC for different tuning parameters, with each point averaged over 100 applications of two-fold cross-validation. The point A reflects the tuning parameter value yielding the maximum AUC, and is depicted in the curves in **(B)**. The traces define a restricted space of tuning parameters. Above and to the right of the white trace yields no more than an average of 75 predictors, and below and to the left of the black trace reflects at least 0.90 AUC on average. **(B)** ROC curve (in black) reflecting high prediction accuracy based on 271 imaging predictors; AUC is 0.989. The colored curves highlight the variability associated with each separate CV sample.

The resulting average AUC values in the (α, λ) grid are shown in Figure 4A. Point A indicates the (α, λ) combination with the maximum average area under the curve, AUC = 0.989. The corresponding average ROC curve (black) is shown in Figure 4B, along with the individual ROC curves from each cross-validation fit, indicating the degree of variability across samples. Point A, at α = 0.02, is very close to ridge regression and, correspondingly, there is only a slight degree of feature selection. The average number of nonzero coefficients over the 200 training samples is 245.3 (out of 271). Moreover, no feature is consistently excluded over the 200 samples. So, while on average the models achieve remarkable accuracy in distinguishing PD patients from healthy controls, the large number of contributing variables involved does not advance our goal of identifying potential biomarkers that can be considered in future research to explore possible biological mechanisms. Therefore, despite attaining high prediction accuracy, our pursuit of potential markers prompts us to seek additional parsimony.

We proceed by constructing a bounded search region, *B* = {(α, λ) | *p* ≤ 75, *AUC* ≥ 0.9}, for the tuning parameters to induce parsimony (see Figure 4A). The white boundary partitions the search region so that the area to the right has, on average, *p* ≤ 75 variables. To the left, the black trace defines the area with average *AUC* ≥ 0.9 to ensure that we retain a sufficient level of accuracy. The operating points between the two lines make up set *B*.

**Figure 5:**
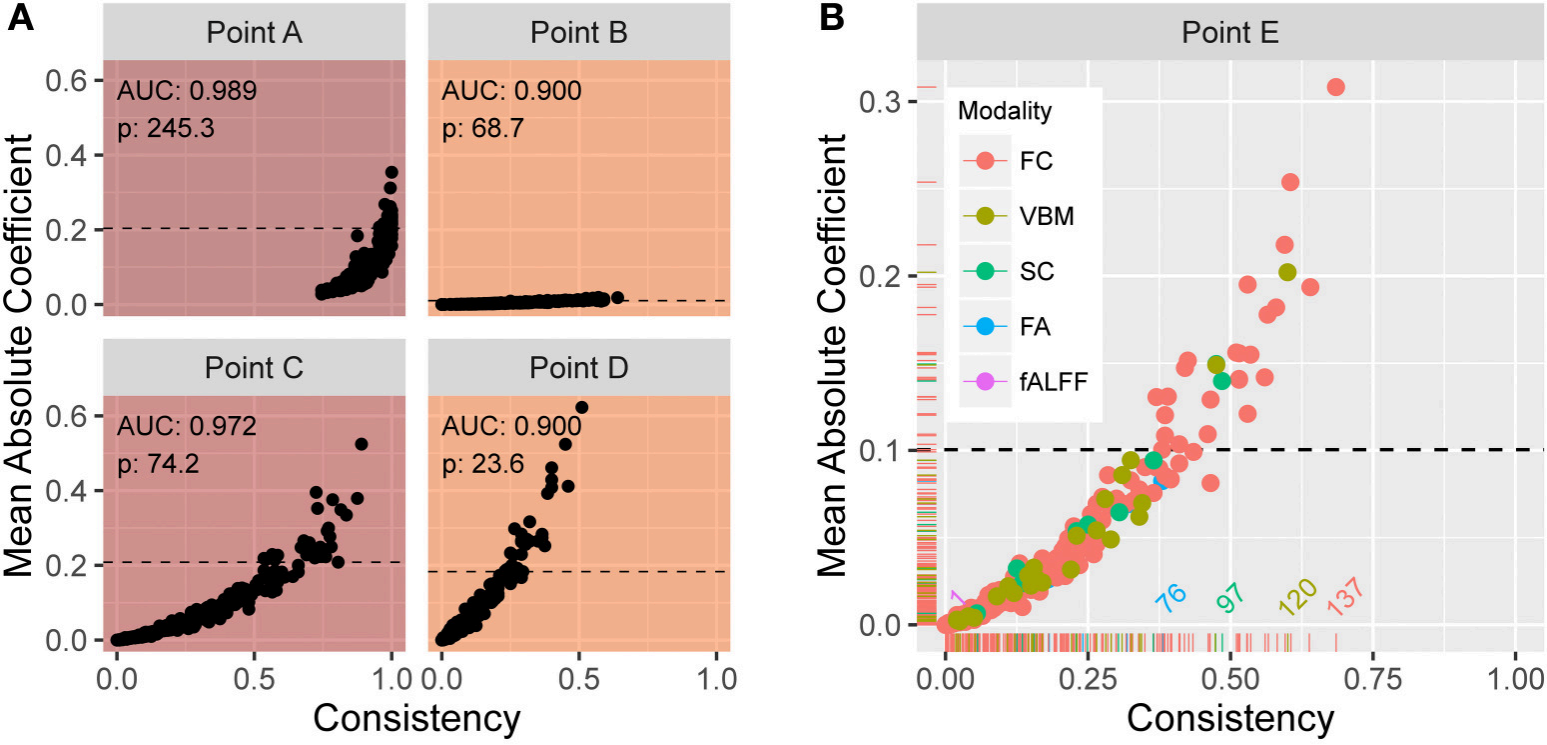
**(A)** Plots of the mean absolute coefficient (standardized) vs. the proportion of times the feature is retained over 200 training samples at (α, λ) corresponding to points (B–E) in Figure 4A. The enlarged plot shown in **(B)** is point E from Figure 4A, with colors depicting the modality. The reference lines in all plots reveal the 10% of values with strongest predictive power over the training samples. At point E, modalities FC, SC, and VBM yield the most predictive features.

From the previously described elastic net with repeated two-fold cross-validation, Figure 5A shows scatter plots of the mean absolute coefficient of each standardized feature vs. the proportion of instances the feature is retained (i.e., has a nonzero coefficient) over the 200 training samples. Each plot corresponds to operating points A, B, C, and D in Figure 4A. At point A, we see that the mean absolute coefficient values are relatively large, and that every feature is selected 75% or more of the 200 trials. Points B, C, and D explore different extremes of our bounded search region. As alpha increases, the rate at which features are selected decreases. At large lambda (point B), the mean coefficient values are small. In each panel, the horizontal line indicates the threshold ξ_0.10_ signifying the top 10% with the strongest predictive features (based on mean absolute coefficient value). Figure 5B shows an enlarged plot at point E, a representative point near the middle of the search region. Using color, the plot illustrates the distribution associated with the different modalities. At point E, modalities FC, SC, and VBM yield the most predictive features.

Considering all operating points in the search area, the resulting set *C* includes 24 features, each of which is consistently among the most predictive for at least 90% (τ_*C*_ = 0.9) of the operating points in *B*. The features are listed in Table 2 and include 21 FC, 1 SC, and 2 regional VBM measures.

**Table 2:** **List of 24 features that are consistently the most predictive across a restricted tuning parameter space for (α, λ) in the elastic net procedure**.

**Feature**	**Upper 10% predictive strength over (α, λ)(%)**	**Upper 10% selection rate over (α, λ)(%)**	**Direction of effect**
FC: Frontal Sup Orb L × Temporal Pole Sup L	94.4	99.4	1
FC: Amygdala R × Angular R^*^	97.5	46.0	1
FC: Amygdala R × Lingual L	100.0	100.0	1
FC: Calcarine L × Thalamus L	100.0	100.0	−1
FC. Cingulum Ant R × Cingulum Post L	100.0	100.0	1
FC: Cuneus R × Precuneus R	100.0	100.0	−1
FC: Frontal Inf Orb R × Temporal Mid R	99.7	90.7	1
FC: Frontal Inf Orb R × Temporal Mid L	100.0	100	1
FC: Frontal Inf Tri R × Temporal Pole Mid R	100.0	100.0	1
FC: Frontal Mid Orb L × Hippocampus L	100.0	100.0	1
FC: Frontal Sup Medial L × Cingulum Ant L	95.2	0.85	1
FC: Frontal Sup Orb L × Insula L	100.0	100.0	1
FC: Frontal Sup Orb L × Parietal Inf L	100.0	100.0	1
FC: Frontal Sup Orb L × Temporal Sup R	100.0	100.0	−1
FC: Occipital Mid L × Occipital Inf R	99.1	98.0	1
FC: Occipital Sup L × Temporal Mid R	100.0	100.0	−1
FC: Occipital Sup R × Precuneus R	100.0	61.2	−1
FC: Temporal Mid R × Temporal Pole Mid R	100.0	100.0	1
FC: Temporal Sup R × Temporal Pole Mid L	100.0	99.7	1
FC: Thalamus L × Temporal Pole Mid L	100.0	100.0	−1
SC: Calcarine L × Precuneus_R	100.0	87.5	−1
VBM: Frontal Inf Orb R	100.0	100.0	−1
VBM: Frontal Mid R	100.0	98.9	−1

Predictive strength, for a given (α, λ), was computed as the mean absolute coefficient (normalized) across 200 training samples. 18 features were retained at 100% of the tuning parameter values considered. The list includes 21 FC features, 2 regional VBM measures, and 1 SC measure.

* Two distinct FC links between these regions.

**Figure 6:**
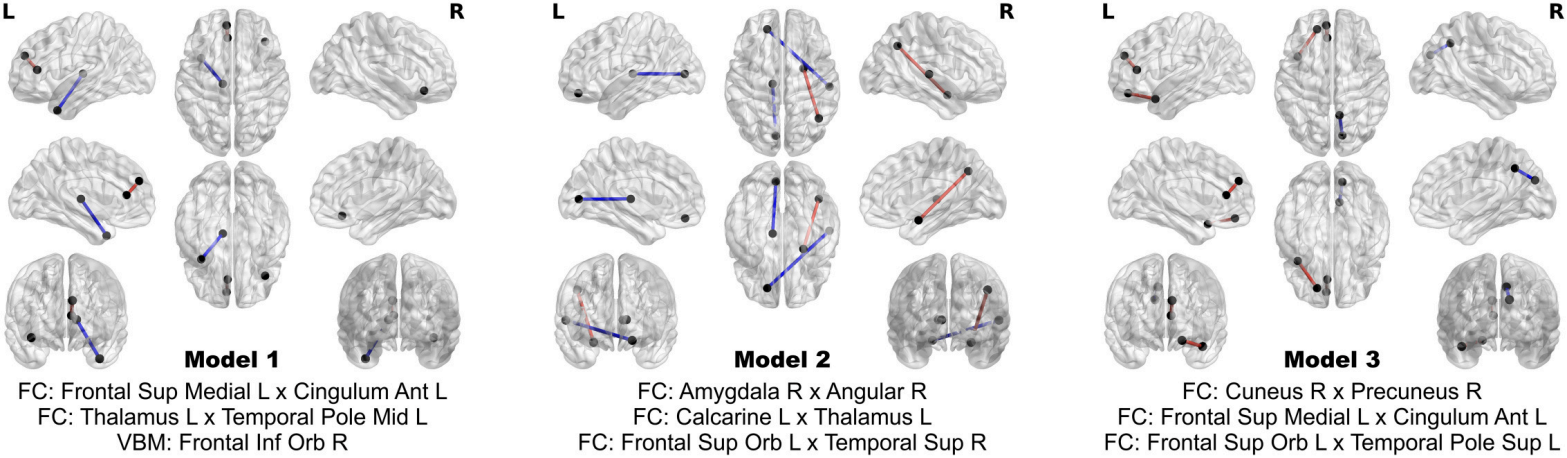
**Models achieving perfect separation between PD patients and HC subjects with a minimum number of variables**. Each three feature model is adjusted for age, sex, and head coil. The models are comprised of eight distinct features.

The 24 features contribute extremely strong predictive power. Using logistic regression, still controlling for head coil, sex, and age, one can achieve perfect separation between PD patients and HC using subsets of as few as three of these multimodal imaging features. In fact, out of all possible three-feature models, three of them achieve perfect separation between the groups, and comprise an aggregate of eight separate features. The three models and the associated map of features are presented in Figure 6. No model of less than three features achieves perfect separation; however many such models exist when more than three out of the 24 features are considered.

Performing univariate screening as a separate step prior to cross validation of the elastic net could potentially impact variable selection and classification performance. To examine this possibility for our analysis, we performed univariate screening using the same 200 (2x100) cross validation training samples constructed in the elastic net stage. Note that for this sensitivity analysis, we did not additionally implement our bootstrap procedure within each of the cross validation samples, given the computational cost. We track the number of times each feature's corresponding *p*-value falls below the designated modality-specific threshold across the 200 samples. Features that pass the threshold in 75% or more of the 200 cross validation samples are regarded to pass univariate screening in our sensitivity analysis.

Nearly all (23 of 24) features that emerged from the elastic net stage in our original analysis were also selected by this revised cross-validation univariate screening. The excluded feature, the functional connectivity between the right frontal inferior orb and the middle temporal lobe, fell just below our threshold by being selected in only 73% of the 2 × 100 cross-validation trials. Thus our findings suggest that the final panel of 24 features is not substantially impacted by the decoupling of screening and cross validation, perhaps buffered by the addition of our bootstrap screening procedure which accounts for sampling variability.

High dimensional prediction and classification methods are subject to inflated measures of accuracy for the particular data set under consideration, resulting in findings that are not reproducible on independent data sets. We take measures to minimize the risk of overfitting and to assess the potential influence of overfitting in our analysis. In particular, we perform iterated two-fold cross-validation in the elastic net procedure with 271 variables as outlined above. To assess the presence and potential influence of overfitting, we conduct a null randomization experiment in which we combine all subjects and then randomly assign the subjects to one of two groups, with the group sizes matched to the actual sizes of the PD and control groups in our experimental data. In our null data, one would not expect to observe systematic between-group differences given the random mixing of PD and control subjects. We repeat our analysis on the null data and, as expected, obtain an ROC curve that roughly tracks a 45° line (chance), giving no indication of inflated accuracy due to our methods.

We note that this null hypothesis verification was performed only within the cross-validated elastic net analysis, using the 271 features that had previously passed screening and hence been deemed to be strongly associated to disease status. Given the massive number of features relative to the number of subjects in the analysis, it is entirely possible that other sets of features could be found, which have high explanatory affinity for random (perhaps meaningless) subject groupings. We assume that the PD (vs. control) labels reflect true manifestation of disease and thus that the identified features are strong candidates for PD biomarkers.

## Discussion

Our analysis provides a broad multimodal view of prevailing alterations in PD, which serve as accurate and reliable predictors. We identify 24 neural manifestations of PD, which contribute extremely strong predictive power in these subjects (Table 2). FC from resting-state fMRI emerges as the most prominent modality. Decreased SC between the left calcarine area and the right precuneus also is an indicator aiding dissociation of PD and HC subjects. VBM calculated from anatomical T1-MRI scans also contributes to accurate prediction, with PD patients revealing reduced volume in the right inferior orbital frontal cortex (OFC) and right middle frontal gyrus. Our findings support a previous report of volumetric changes in gray matter associated with PD, including bilateral OFC and the right inferior frontal gyrus (rIFG) (Xia et al., 2013) and are consistent with reports of cortical thinning in PD (without dementia) in the middle frontal gyrus and other regions including inferior and superior parietal areas, superior frontal, superior temporal, precuneus, pre- and postcentral, and fusiform regions (Zhang et al., 2015).

Bilaterally, the middle temporal pole (MTP) exhibited strong discriminatory power and consistency. For PD patients, the right MTP shows increased FC with the rIFG pars triangularis (rIFG-PT) and with the right middle temporal gyrus (MTG). The left MTP exhibits decreased FC with the left thalamus in PD patients and increased FC the right superior temporal sulcus (STS). The OFC, which is linked to inhibitory control, exhibits functional connections with several regions that are predictive of PD. Right inferior areas of the OFC show increased FC bilaterally with the MTG. The left middle areas of the OFC show increased FC with the left hippocampus. The left superior OFC exhibits increased FC in PD patients with the left insula, the left inferior parietal region, and the left superior temporal pole; and decreased FC with the right STS. Our analysis also reveals increased FC between the left medial superior frontal gyrus, which includes the supplementary motor area (SMA) and the preSMA, and the left ACC. These FC alterations all yield strong power to dissociate PD patients from controls.

PD symptoms have been linked to FC between the pars triangularis and the orbito-frontal cortex, specifically with FC shown to be positively associated with the Movement Disorder Society (MDS) Unified Parkinson's Disease Rating Scale (UPDRS), part II, entitled Motor Aspects of Experiences of Daily Living (Yoo et al., 2015). Also, some of the OFC functional connections map anatomical tracts known from macaque monkey studies between the orbitofrontal cortex and limbic areas including insular cortex and the hippocampus (Cavada et al., 2000).

Discriminatory power is also drawn from decreased FC in PD patients between the left calcarine and thalamus and between the right cuneus and precuneus as well as increased FC for PD patients between right anterior cingulate cortex (ACC) and the posterior cingulate cortex (PCC) and between the right amygdala and both the left lingual gyrus and the right angular gyrus. In contrast to our work examining specific connections between pairs of brain regions, graph-theoretic approaches seek to characterize whole brain topological properties of brain networks (Simpson et al., 2013). In this complementary view, Göttlich et al. (2013) show that the degree of whole-brain connectivity was decreased in the occipital lobe (cuneus and calcarine), but increased in the superior parietal cortex, PCC, supramarginal gyrus and supplementary motor area.

Several of the above regions have been identified for PD-related alterations or dysfunction. The amygdala plays a key role in memory, decision-making, and emotional response. The amygdala may undergo a loss of gray-matter volume in PD due to neurodegeneration (Harding et al., 2002). Hu et al. (2015) found that, relative to healthy controls, depressed PD patients exhibited decreased right amygdala FC with the left gyrus rectus, left inferior OFC, and right putamen. The FC alterations in the amygdala may be driven by the severe pathological changes that occur in this region and the major projections to the prefrontal cortex and limbic system (hippocampus and entorhinal region), among others (Braak et al., 1994). Van Eimeren et al. (2009) detected different deactivation patterns in the PCC and the precuneus in PD patients relative to healthy controls.

Our analysis considers extremely high-dimensional data and eventually selects a small number of variables, representing just 0.051% of the original features considered, with subsets representing 0.0064% of the features achieving perfect separation of the PD patients and the HC subjects. An important step in the route to developing reliable biomarkers is to validate the identified features in independent data sets. We take many steps to encourage reproducibility here within our sample, but ultimately adoption of biomarkers requires external validation. It is likely that many other useful predictors are present in the data, so our results do not preclude the possibility that important predictive information may be gleaned from a neuroimaging modality/feature that was ultimately excluded from our final model. Moreover, our data representations may have excluded potentially useful markers. For example, we focused our analysis on AAL ROIs, opting to maintain consistency of the regions across modalities (with possibly nested subregions). AAL regions are predominantly composed of gray matter and contain relatively less white matter (median across regions and subjects is roughly 15%). As such, we may have excluded potentially predictive markers from DTI-related measures (FA or SC) sampled from regions dominated by white matter. Also, the features extracted from the multimodal imaging data reflect particular characteristics at a selected spatial scale. Some data reduction is necessary, e.g., to limit the data from generating billions of features. We cannot determine in advance, which spatial scale will extract maximal information for the purpose of dissociating PD patients from controls.

## Author contributions

FB conceptualized methodology and guided evolution of the research. FB also wrote the majority of the paper. DD processed the imaging data and performed statistical analyses on the results. DD contributed to the data and methods section and generated tables and figures for the paper. DH managed data collection and provided expertise in Parkinson's disease research and imaging. DH provided thoughtful comments and corrections throughout the paper.

### Conflict of interest statement

The authors declare that the research was conducted in the absence of any commercial or financial relationships that could be construed as a potential conflict of interest.

## References

[B1] Andersson J.L. R. Skare S. Ashburner J. (2003). How to correct susceptibility distortions in spin-echo echo-planar images: application to diffusion tensor imaging. Neuroimage 20, 870–888. 10.1016/S1053-8119(03)00336-7 14568458

[B2] Behrens T. E. J. Johansen-Berg H. Jbabdi S. Rushworth M. F. S. Woolrich M. W. (2007). Probabilistic diffusion tractography with multiple fibre orientations. What can we gain? Neuroimage 23, 144–155. 10.1016/j.neuroimage.2006.09.018 17070705 PMC7116582

[B3] Braak H. Braak E. Yilmazer D. de Vos R. A. Jansen E. N. Bohl J.. (1994). Amygdala pathology in Parkinson's disease. Acta Neuropathol. 88, 493–500. 10.1007/BF00296485 7879596

[B4] Braak H. Del Tredici K. Rüb U. de Vos R. A. Jansen Steur E. N. Braak E. (2003). Staging of brain pathology related to sporadic Parkinson's disease. Neurobiol. Aging 24, 197–211. 10.1016/S0197-4580(02)00065-9 12498954

[B5] Cavada C. Company T. Tejedor J. Cruz-Rizzolo R. J. Reinoso-Suarez (2000). The anatomical connections of the macaque monkey orbitofrontal cortex. Cereb. Cortex 10, 220–242. 10.1093/cercor/10.3.220 10731218

[B6] Cox R. W. (1996). AFNI: Software for analysis and visualization of functional magnetic resonance neuroimages. Comput. Biomed. Res. 29, 162–173. 10.1006/cbmr.1996.0014 8812068

[B7] Del Tredici K. Braak H. (2013). Dysfunction of the locus coeruleus-norepinephrine system and related circuitry in Parkinson's disease-related dementia. J. Neurol. Neurosurg. Psychiatry 84, 774–783. 10.1136/jnnp-2011-301817 23064099

[B8] Du G. Lewis M. M. Styner M. Shaffer M. L. Sen S. Yang Q. X.. (2011). Combined R2^*^ and diffusion tensor imaging changes in the substantia nigra in Parkinson's disease. Mov. Disord. 26, 1627–1632. 10.1002/mds.23643 21618607 PMC3154471

[B9] Fearnley J. M. Lees A. J. (1991). Ageing and Parkinson's disease: substantia nigra regional selectivity. Brain 114, 2283–2301 1933245 10.1093/brain/114.5.2283

[B10] Fiandaca M. S. Mapstone M. E. Cheema A. K. Federoff H. J. (2014). The critical need for defining preclinical biomarkers in Alzheimer's disease. Alzheimers Dement. 10(Suppl. 3), 196–212. 10.1016/j.jalz.2014.04.015 24924671

[B11] Foroud T. Smith D. Jackson J. Verbrugge J. Halter C. Wetherill L.. (2015). Novel recruitment strategy to enrich for LRRK2 mutation carriers. Mol. Genet. Genomics Med. 3, 404–412. 10.1002/mgg3.151 26436106 PMC4585448

[B12] Gaser C. (2010). Author Archive for Christian Gaser. Structural Brain Mapping Group. Available online at: http://www.neuro.uni-jena.de/vbm/

[B13] Göttlich M. Münte T. F. Heldmann M. Kasten M. Hagenah J. Krämer U. M. (2013). Altered resting state brain networks in Parkinson's disease. PLoS ONE 8:e77336. 10.1371/journal.pone.0077336 24204812 PMC3810472

[B14] Harding A. J. Stimson E. Henderson J. M. Halliday G. M. (2002). Clinical correlates of selective pathology in the amygdala of patients with Parkinson's disease. Brain 125, 2431–2445. 10.1093/brain/awf251 12390970

[B15] Hu X. Song X. Yuan Y. Li E. Liu J. Liu W.. (2015). Abnormal functional connectivity of the amygdala is associated with depression in Parkinson's disease. Mov. Disord. 30, 238–244. 10.4137/JEN.S33444 25545969

[B16] Iranzo A. Tolosa E. Gelpi E. Molinuevo J. L. Valldeoriola F. Serradell M.. (2013). Neurodegenerative disease status and post-mortem pathology in idiopathic rapid-eye-movement sleep behaviour disorder: an observational cohort study. Lancet Neurol. 12, 443–453. 10.1016/S1474-4422(13)70056-5 23562390

[B17] Kahan J. Urner M. Moran R. Flandin G. Marreiros A. Mancini L.. (2014). Resting state functional MRI in Parkinson's disease: the impact of deep brain stimulation on ‘effective’ connectivity. Brain 137, 1130–1144. 10.1093/brain/awu027 24566670 PMC3959559

[B18] Kalia L. V. Lang A. E. (2015). Parkinson's disease. Lancet 386, 892–912. 10.1016/s0140-6736(14)61393-3 25904081

[B19] Lee E. Y. Sen S. Eslinger P. J. Wagner D. Shaffer M. L. Kong L.. (2013). Early cortical gray matter loss and cognitive correlates in non-demented Parkinson's patients. Parkinsonism Relat. Disord. 19, 1088–1093. 10.1016/j.parkreldis.2013.07.018 23932064 PMC3858507

[B20] Parkinson's Disease Foundation (2015). Statistics on Parkinson's. Available online at: http://www.pdf.org/en/parkinson_statistics

[B21] Postuma R. B. Iranzo A. Hogl B. Arnulf I. Ferini-Strambi L. Manni R.. (2015). Risk factors for neurodegeneration in idiopathic rapid eye movement sleep behavior disorder: a multicenter study. Ann. Neurol. 77, 830–839. 10.1002/ana.24385 25767079 PMC5769479

[B22] Simpson S. L. Bowman F. D. Laurienti P. J. (2013). Analyzing complex functional brain networks: fusing statistics and network science to understand the brain. Stat. Surv. 7, 1–36. 10.1214/13-SS103 25309643 PMC4189131

[B23] Smith S. M. Jenkinson M. Woolrich M. W. Beckmann C. F. Behrens T. E. J. Johansen-Berg H.. (2004). Advances in functional and structural MR image analysis and implementation as FSL. Neuroimage 23, 208–219. 10.1016/j.neuroimage.2004.07.051 15501092

[B24] Strimbu K. Tavel J. A. (2010). What are Biomarkers? Curr. Opin. HIV AIDS 5, 463–466. 10.1097/COH.0b013e32833ed177 20978388 PMC3078627

[B25] Tolosa E. Pont-Sunyer C. (2011). Progress in defining the premotor phase of Parkinson's disease. J. Neurol. Sci. 310, 4–8. 10.1016/j.jns.2011.05.027 21679972

[B26] Tzourio-Mazoyer N. Landeau B. Papathanassiou D. Crivello F. Etard O. Delcroix N.. (2002). Automated anatomical labeling of activations in SPM using a macroscopic anatomical parcellation of the MNI MRI single-subject brain. Neuroimage 15, 273–289. 10.1006/nimg.2001.0978 11771995

[B27] Vaillancourt D. E. Spraker M. B. Prodoehl J. Abraham I. Corcos D. M. Zhou X. J.. (2009). High-resolution diffusion tensor imaging in the substantia nigra of de novo Parkinson disease. Neurology 72, 1378–1384. 10.1212/01.wnl.0000340982.01727.6e 19129507 PMC2677508

[B28] Van Eimeren T. Monchi O. Ballanger B. Strafella A. P. (2009). Dysfunction of the default mode network in Parkinson disease: a functional magnetic resonance imaging study. Arch. Neurol. 66, 877–883. 10.1001/archneurol.2009.97 19597090 PMC2972248

[B29] Xia J. Wang J. Tian W. Ding H. Wei Q. Huang H.. (2013). Magnetic resonance morphometry of the loss of gray matter volume in Parkinson's disease patients. Neural Regen. Res. 8, 2557–2565. 10.1371/journal.pone.0131133 25206566 PMC4145936

[B30] Yoo K. Chung S. J. Kim H. S. Choung O.-H. Lee Y. B. Kim M. J.. (2015). Neural substrates of motor and non-motor symptoms in Parkinson's disease: a resting fMRI study. PLoS ONE 10:e0125455. 10.1371/journal.pone.0125455 25909812 PMC4409348

[B31] Zarei M. Ibarretxe-Bilbao N. Compta Y. Hough M. Junque C. Bargallo N.. (2013). Cortical thinning is associated with disease stages and dementia in Parkinson's disease. J. Neurol. Neurosurg. Psychiatry 84, 875–881. 10.1007/s00429-014-0785-x 23463873 PMC3717586

[B32] Zhang L. Wang M. Sterling N. Lee E. Eslinger P. Wagner D. (2015). Cortical thinning and cognitive impairment in Parkinson's disease without dementia, in IEEE/ACM Transactions on Computational Biology and Bioinformatics. 10.1109/TCBB.2015.2465951 PMC5918696 29610105

[B33] Zou H. Hastie T. (2005). Regularization and variable selection via the elastic net. J. R. Stat. Soc. B 67, 301–320. 10.1111/j.1467-9868.2005.00503.x

[B34] Zou Q. H. Zhu C. Z. Yang Y. Zuo X. N. Long X. Y. Cao Q. J.. (2008). An improved approach to detection of amplitude of low-frequency fluctuation (ALFF) for resting-state fMRI: fractional ALFF. J. Neurosci. Methods 172, 137–141. 10.1016/j.jneumeth.2008.04.012 18501969 PMC3902859

